# miRNA–lncRNA Cross-Regulation Landscape in Cancer: From Molecular Mechanisms to Therapeutic and Diagnostic Applications

**DOI:** 10.3390/cancers18101610

**Published:** 2026-05-15

**Authors:** Giuseppe Scafuro, Myriam Karam, Ayesha Khan, Chiara Tammaro, Takehiro Nagatsuka, Anna Grimaldi, Alessia Maria Cossu, Silvia Zappavigna, Michele Caraglia, Gabriella Misso, Michela Falco

**Affiliations:** 1 Department of Precision Medicine, University of Campania “Luigi Vanvitelli”, Via L. De Crecchio, 7, 80138 Naples, Italy; myriam.karam@unicampania.it (M.K.); ayesha.khan@unicampania.it (A.K.); chiara.tammaro@unicampania.it (C.T.); takehiro.nagatsuka@unicampania.it (T.N.); michele.caraglia@unicampania.it (M.C.); michela.falco@unicampania.it (M.F.); 2 Sylvester Comprehensive Cancer Center, Miller School of Medicine, University of Miami, Miami, FL 33136, USA; 3 Drug Discovery Laboratory, Wakunaga Pharmaceutical Co., Ltd., 1624 Shimokotachi, Koda-Cho, Akitakata-Shi, Hiroshima 739-1195, Japan; 4 U.P. Cytometric and Mutational Diagnostics, Vanvitelli Hospital, University of Campania “Luigi Vanvitelli”, 83031 Naples, Italy; grim.anna@tiscali.it; 5 Laboratory of Molecular and Precision Oncology, BIOGEM Scarl, Institute of Genetic Research, 83031 Ariano Irpino, Italy

**Keywords:** microRNAs (miRNAs), long non-coding RNAs (lncRNAs), competing endogenous RNA (ceRNA), cancer biology, biomarkers, RNA therapeutics, miRNA mimics, antagomiRs, multi-omics, network biology

## Abstract

**Background/Objectives:** Over the past two decades, non-coding RNAs (ncRNAs) have emerged as key regulators of gene expression, reshaping the classical view of the genome as predominantly protein-coding. Among them, microRNAs (miRNAs) and long non-coding RNAs (lncRNAs) play central roles in controlling gene expression at multiple levels. Rather than acting independently, these molecules form complex and interconnected regulatory networks, and their interplay appears particularly relevant in cancer. This review aims to examine the mechanisms underlying miRNA-lncRNA cross-regulation and to explore their functional and clinical implications in tumor biology. **Methods:** We performed a comprehensive analysis of the current literature focusing on studies investigating miRNA-lncRNA interactions in cancer. Particular attention was given to mechanistic insights, including the competing endogenous RNA (ceRNA) hypothesis, as well as alternative regulatory models involving direct RNA interactions and chromatin-associated processes. **Results:** miRNA-lncRNA interactions have been associated with cancer progression and therapeutic response across different tumor types, although their mechanisms are highly context-dependent. While the ceRNA hypothesis, based on competition for shared microRNA response elements (MREs), provides a useful framework, it does not fully explain all observed phenomena. Evidence shows that miRNAs can directly regulate lncRNA stability, whereas lncRNAs can influence miRNA biogenesis. Additionally, chromatin-related mechanisms suggest that these interactions extend beyond post-transcriptional regulation. These RNA networks intersect with major oncogenic pathways, including PI3K/AKT/mTOR signaling, hypoxia responses, and epigenetic regulators such as EZH2, thereby affecting key cancer processes such as proliferation, epithelial–mesenchymal transition (EMT), and metabolic reprogramming. From a clinical perspective, the stability of ncRNAs in biological fluids highlights their potential as biomarkers. Combined miRNA-lncRNA signatures may improve diagnostic and prognostic accuracy compared to single markers, although further validation is required. Therapeutic strategies targeting ncRNA networks, such as miRNA mimics, antagomiRs, and lncRNA-directed approaches, are under investigation; however, challenges related to delivery, specificity, and toxicity remain. **Conclusions:** miRNA-lncRNA cross-regulation represents a complex and multifaceted layer of gene regulation in cancer. A deeper understanding of these interactions could support the development of more accurate diagnostic tools and more effective RNA-based therapeutic strategies, although significant technical and biological challenges still need to be addressed.

## Simple Summary

Non-coding RNAs, particularly microRNAs (miRNAs) and long non-coding RNAs (lncRNAs), are key regulators of gene expression that interact within complex networks and play a central role in cancer. Their cross-regulation influences major cellular processes such as proliferation, epithelial–mesenchymal transition, and metabolism, and is linked to important oncogenic pathways. While the ceRNA hypothesis partially explains these interactions, additional mechanisms, including effects on RNA stability, biogenesis, and chromatin regulation, also play significant roles. In this context, computational and systems biology approaches are increasingly important for integrating large-scale datasets and reconstructing these networks, helping to identify functional interactions and clinically relevant signatures. Due to their stability in biological fluids, combined miRNA-lncRNA signatures show promise as diagnostic and prognostic biomarkers, and targeting these networks also offers new therapeutic opportunities.

## 1. Introduction

Over many years, the scientific community has mostly exclusively described and studied protein-coding regions of the human genome. After the launch of the ENCODE Project (2003) and the subsequent completion of the human genome sequencing, it was discovered that less than 2% of the genome encodes proteins, while the vast majority of it is actively transcribed into non-coding RNAs (ncRNAs). These transcripts, originating from regions initially defined as “junk DNA” play a fundamental role in the sophisticated regulation of cellular identity and homeostasis [1]. Particularly in the highly complex context of malignancy, dysregulation of the non-coding DNA landscape not only promotes genetic instability but is also a key factor in tumorigenesis, metastasis, and therapy resistance [2]. Given the importance of ncRNAs, especially microRNA (miRNA) and long ncRNA (lncRNA), and their interactions in different types of cancer, understanding their functional differences and regulatory mechanisms is essential for deciphering the cancer transcriptome and developing potential therapeutic applications [3].

Non-coding RNAs exhibit different lengths as well as remarkable functional versatility. MiRNAs act primarily as post-transcriptional regulators, specializing in gene silencing and functioning either as oncomiRs or tumor suppressors, based on the cellular context and their targets [4]. In contrast, lncRNAs act at both transcriptional and post-transcriptional levels to regulate gene expression. Their structural complexity makes them similar to proteins; through the formation of secondary and tertiary structures, lncRNAs can interact with proteins and nucleic acids such as DNA and RNA [5]. In this scenario, the discovery of competitive endogenous RNAs (ceRNAs) has transcended the classical view of RNA as a passive messenger. Salmena et al. first proposed the hypothesis of ceRNA as a complex network where lncRNAs, circRNAs, and mRNAs sharing common microRNA response elements (MREs) compete for a limited pool of miRNAs. In this regulatory circuit, lncRNAs can act as molecular sponges, sequestering miRNAs and preventing their binding to target mRNAs. In cancer, lncRNA overexpression can effectively silence tumor-suppressive miRNAs, resulting in the upregulation of oncogenes. This regulatory network adds an additional layer of complexity beyond traditional linear signaling pathways [6].

Several molecular and spatial variables may play a critical role in determining the effectiveness of crosstalk within the ceRNA network [6]. First and foremost, the abundance and affinity of miRNA MRE regions influence the ability of an lncRNA to bind and sequester miRNAs. The strength of this interaction depends on the number of binding sites on the lncRNA sequence and the thermodynamic stability of the resulting miRNA-lncRNA duplex [7]. Greater sequence complementarity generally results in more stable binding, allowing the lncRNA to function as a more efficient molecular “sponge” [8]. A growing body of evidence has described ceRNA crosstalk as a cytoplasmic phenomenon; however, emerging studies also reported nuclear crosstalk, in which lncRNA-miRNA interactions overcome the canonical silencing function by influencing several processes, such as the recruitment of splicing factors or chromatin modifiers, thereby altering epigenetic states [9]. The balance of the entire network is governed by the stoichiometric relationship between the main components: the “sponge” (lncRNA), the “target” (mRNA) and the “mediator” (miRNA). If the ratio among the concentrations of lncRNA, miRNA and its natural targets is not maintained, particularly if the concentration of lncRNA is not sufficiently high relative to the others, the competitive effect may become functionally negligible, leading to disruption of this delicate balance and affecting the broader cellular regulatory network [10]. In cancer, the balance of the ceRNA network is frequently disrupted by molecular alterations that contribute to tumor progression. In solid tumors, for example, the overexpression of specific lncRNAs, in particular MALAT1 and H19, is one of the main oncogenic factors [11,12]. These lncRNAs can function as competing endogenous RNAs by interacting with tumor-suppressive miRNAs, such as members of the let-7 or miR-200 families, thereby reducing their availability for canonical mRNA targets. lncRNAs may also indirectly increase the expression of oncogenic mRNAs, thereby promoting key processes such as epithelial–mesenchymal transition (EMT) and metastatic dissemination [13,14].

These regulatory networks can be explored using computational approaches. Several algorithms based on sequence complementarity, including TargetScan, miRanda, and StarBase, enable the prediction of potential miRNA–lncRNA interactions. Future progress in this field will likely rely on integrative omics approaches: the combination of RNA-seq data with CLIP-seq (Cross-Linking ImmunoPrecipitation) will enable the experimental validation of miRNAs that are actually sequestered by specific lncRNAs in particular neoplastic contexts [6,15].

It is therefore clear that identifying interactions between ncRNAs is fundamental for the advancement of precision medicine. Among ncRNAs, miRNAs and lncRNAs are increasingly assuming a central role in liquid biopsy. Owing to their high stability in biological fluids, resulting from encapsulation in exosomes or association with protective proteins such as Argonaute proteins, they have emerged as promising biomarkers [16,17]. The clinical use of ncRNAs promotes early and non-invasive diagnosis, offers a dynamic tool for monitoring therapeutic response and the onset of chemoresistance in real time, and opens new therapeutic perspectives [18,19]. The reversible nature of their interactions effectively makes the use of antagomiRs or miRNA mimics capable of pharmacologically modulating the ceRNA network, thereby representing one of the most advanced frontiers in molecular oncology [20,21].

In conclusion, the identification and study of ceRNA cross-talk is useful for mapping the complexity of the non-coding genome and, above all, for the development of innovative diagnostic and therapeutic approaches capable of interfering with tumor cell regulatory mechanisms.

In this review, we provide an overview of the emerging role of lncRNA–miRNA crosstalk within the ceRNA network in cancer. We will focus on the molecular mechanisms regulating these interactions, their computational identification and we will discuss their importance as potential biomarkers and/or target therapy in precision oncology.

## 2. Overview of miRNAs and lncRNAs in Cancer

Modern molecular biology has transitioned from the concept of non-functional DNA to that of a regulatory ecosystem, placing ncRNAs at the forefront of oncology research. miRNAs and lncRNAs, specifically, have emerged as the main modulators of oncogenic mechanisms from neoplastic transformation to progression and distant diffusion, as demonstrated in several tumor types [22,23]. In the field of liquid biopsy, the stability of ncRNAs in biological fluids is becoming more crucial. Detection of specific molecular patterns of miRNAs or lncRNAs in patient fluids [24] allows for the real-time monitoring of tumor and treatment response. In addition, ncRNAs’ specificity in the clinical setting makes them a promising therapeutic approach.

### 2.1. MicroRNAs

MicroRNAs (miRNAs) are a group of non-coding small RNA molecules that are conserved through evolution and typically have a length of 18–22 nucleotides. miRNAs impact gene expression by binding to 3′-untranslated regions (3′-UTRs) of target messenger RNAs (mRNAs), promoting translational repression or mRNA degradation [25]. MiRNA biogenesis is tightly controlled and can be broken down into three phases: transcription in the nucleus, translocation between nucleus and cytoplasm, and maturation in the cytoplasm [26]. RNA Polymerase II (Pol II) or RNA Polymerase III (Pol III) is responsible for generating the primary miRNA (pri-miRNA) during nuclear transcription. This transcript, which is several hundred nucleotides long, has a hairpin stem with 33 base-paired regions, a terminal loop, and two single-stranded flanking regions both up and downstream of the hairpin stem [27,28]. In the following step, each pri-miRNA can be recognized and cleaved by the microprocessor complex, producing a ~70-nucleotide precursor hairpin known as pre-miRNAs (pre-miRNA). The core of the nuclear microprocessor complex is represented by RNase III enzyme Drosha and the RNA-binding protein DGCR8 [29]. Then, Exportin-5 transports pre-miRNA from the nucleus to the cytoplasm where DICER1 further cleaves the hairpin precursor close to the terminal loop sequence, producing an miRNA duplex consisting of roughly 22 nucleotides with two nucleotides at each 3’-end. It is theoretically possible to generate two different strands based on the thermodynamic stability of the base pairs at the two ends of the miRNA duplex. The guide strand, with the less stable base pair at its 5’-end, associates with Argonaute (AGO) proteins to induce gene silencing through miRNA-induced silencing complex (miRISC). At this stage, post-transcriptional regulation of gene expression occurs due to the complementarity between the miRNA seed sequence and the target messenger’s 3’UTR region. Perfect complementarity results in endonucleotide cleavage, whereas partial complementarity triggers translational inhibition and deadenylation [30]. In the past two decades, the pathogenesis of many human diseases, including cancer, has been widely shown to be linked to miRNA dysregulation. Di Leva G (2014) asserts that the presence of miRNAs that are not expressed normally plays an important role in both the genesis and progression of human malignancies [31]. MiRNAs have the potential to function as tumor suppressors or oncogenes depending on their target genes [32].

MiRNAs known as OncomiR are up-regulated in cancer cells and can be responsible for carcinogenesis by regulating tumor-suppressor genes [33]. The miR-17-92 cluster, which is found on chromosome 13q31 and is amplified in lung cancer and various lymphomas, is a prominent example [34]. Hayashita Y, 2005, found that this cluster is associated with oncogenic functions and significant over-expression of its members is observed especially in the most aggressive forms of the tumor [35]. Furthermore, it was proven that the miR-17-92 cluster is able to boost proliferation, angiogenesis, and cell survival while also controlling E2F, PTEN, p21, and BIM [36]. Similarly, the leukemogenesis in CLL is caused by the overexpression of miR-155 which down-regulates SHIP1 and disrupts B-cell receptor signaling [37]. In addition, miR-155 expression was found to be abnormal in Hodgkin lymphoma, primary mediastinal and diffuse Large-B-cell lymphoma, as well as solid tumors like breast cancer [38,39,40].

In contrast, tumor-suppressor miRNAs are often downregulated in cancer. Inhibiting certain oncogenes and/or genes involved in apoptosis or cell differentiation control is how they typically prevent tumor development [33]. The highly conserved human let-7 family is a well-known example of tumor-suppressor miRNAs, which are frequently deleted in cancer [41]. The decrease in let-7 expression in lung cancer cells was discovered by Takamizawa and collaborators, which was strongly correlated with less favorable postoperative outcomes [42]. Let-7 acts by silencing powerful oncogenes such as RAS, MYC e HMGA2. Recent studies have analyzed RAS regulation emphasizing that let-7’s tumor-suppressor function is confirmed by its significant underexpression with increased RAS levels in lung cancer, while under normal conditions it negatively regulates RAS [43]. MiR-34a is another miRNA that has been well-characterized as a tumor suppressor in a variety of tumors, such as multiple myeloma (MM), lung cancer, breast cancer, liver cancer, colorectal cancer, prostate cancer, osteosarcoma, CLL and acute myeloid leukemia (AML) [44,45]. p53 can directly bind to the miR-34a promoter in a positive feedback loop to trigger apoptosis in miR-34a [46]. In turn, miR-34a is able to increase the tumor-suppressor activity of p53 by targeting SIRT1, a negative regulator of p53 via deacetylation, increasing p53 tumor-suppressor activity [47]. In fact, miR-34a down-regulation affects cell-cycle arrest, cell senescence and apoptosis in cancer cells contributing to chemoresistance [44,48].

### 2.2. Long Non-Coding RNAs (lncRNAs)

LncRNAs, which are single strands of non-protein-coding RNA, usually have a length of over 200 nucleotides. The linear action of miRNAs is not present in lncRNAs because of their complex secondary and tertiary structures, which enable them to interact with DNA, RNA, and proteins simultaneously. In this way, they perform the role of true molecular architects by contributing to the creation of modular scaffolds [49].

RNA polymerase II is the most common transcription enzyme for lncRNAs, and they are typically processed through the following steps: 5’ capping, splicing, and 3’ polyadenylation. Their genomic orientation relative to protein-coding genes is the basis for their classification [50]. LincRNAs (long intergenic non-coding RNAs) are among these, located in intergenic regions. The transcription of lincRNAs occurs from sequences between two coding genes that are not directly related to protein genes. The opposite strand of a coding gene is the transcriptional site for antisense lncRNAs. The overlap between antisense lncRNA and gene regions such as exons or introns can lead to potential regulatory interactions with the corresponding transcript. Thirdly, there are intronic lncRNAs that reside exclusively within the introns of a host gene [51,52]. Lastly, among the diverse groups of lncRNAs, there are circular RNAs (circRNAs). These are a subtype of lncRNA that are produced by back-splicing events, where the ends of the transcript bond to form a loop structure that is covalently closed. The absence of free ends makes circRNAs unique in that they cannot be degraded by exonuclease and therefore accumulate in the cytoplasm [53].

Four fundamental functional strategies are responsible for mediating the main biological effects of lncRNAs. One common mechanism is that of “decoys”: the role of lncRNAs is to sequester miRNAs or transcription factors and stop them from binding to their targets by acting as ‘molecular sponges’. For example, by sponging let-7, the lncRNA H19 indirectly upregulates let-7 targets, thereby promoting tumorigenesis [14]. Another functional modality is that of “scaffolds”, in this case lncRNAs function as scaffolds to put together multiprotein complexes. HOTAIR acts as a platform for connecting Polycomb Repressive Complex 2 (PRC2) and the LSD1 complex. H3K27 methylation and H3K4 demethylation are induced by this complex structure, which silences gene clusters with tumor-suppressor functions [54]. LncRNA can also function as “guides”; they aid in the positioning of chromatin-modifying proteins at the level of specific DNA sequences either close to transcription sites (in cis) or far from them (in trans) [55,56]. A final functional archetype for the regulatory mechanisms controlled by lncRNAs is that of “enhancer lncRNAs” or “eRNAs”. The production of eRNAs by active enhancer regions leads to the stabilization of DNA loops between enhancers and promoters, ultimately leading to the transcription of nearby oncogenes [57]. From a clinical perspective, rather than a mechanistic one, another striking example of lncRNA is represented by MALAT1. MALAT1 plays a critical role in regulating alternative splicing processes and nuclear organization [58,59]; it has been well documented that its overexpression has a negative impact on survival and correlates positively with the high metastatic potential in various neoplastic forms [60]. PCA3 is another prominent clinical example. The overexpression of Pca3 in prostate cancer cells makes it an effective diagnostic biomarker, with a urine dosage that is more specific than traditional PSA [61,62].

Currently, the revolutionary concept of a ceRNA network is attracting increasing interest in the field of ncRNAs. It suggests an intricate network of interconnections among different RNA species, whose disruption has been seen as a hallmark of complex metabolic and oncogenic changes in tumor cells [6,21].

## 3. Molecular Basis of miRNA–lncRNA Cross-Regulation

### 3.1. Competing Endogenous RNA (ceRNA) Networks

MicroRNAs (miRNAs) and long non-coding RNAs (lncRNAs) are widely known to act as regulators of gene expression and are deeply involved in the mechanisms underlying cancer progression. In recent years, lncRNAs have become one of the main objects of research in molecular biology and medicine due to their fundamental regulatory functions. Numerous lncRNAs have been identified as being aberrantly expressed in a wide range of cancer types, where they can function as either oncogenic drivers or tumor suppressors, depending on the cellular context [4,63,64].

Emerging evidence indicates that lncRNAs such as H19, MALAT1, HOTAIR and DANCR interact with several miRNAs, including miR-200a, miR-138, miR-29a-3p, miR-124, miR-130a and miR-33b, thereby forming regulatory networks that modulate tumor cell proliferation, invasion and epithelial–mesenchymal transition (EMT) through ceRNA mechanisms and direct post-transcriptional regulation [65,66,67,68,69].

A recent study by Bocchetti M. et al. demonstrated the existence of a functional regulatory interaction between miR-423-5p and the lncRNA MALAT1 in hepatocellular carcinoma (HCC).

Analysis of HCC patient datasets revealed that high expression of miR-423-5p is associated with a less aggressive tumor phenotype and improved overall and recurrence-free survival, whereas high levels of MALAT1 correlate with poor prognosis. A direct binding site between miR-423-5p and the MALAT1 transcript was identified through bioinformatics analyses combined with experimental validation, suggesting a post-transcriptional regulatory mechanism. The physical association between miR-423-5p and MALAT1 was further confirmed by dual-luciferase reporter assays and AGO2-RNA immunoprecipitation. These results were validated in different models of HCC, supporting the clinical relevance of this miRNA-lncRNA axis [11].

In non-small-cell lung cancer (NSCLC), Wu J. et al. showed that MALAT1 is upregulated in tumor tissues and functions as a molecular sponge for miR-124. Through this ceRNA mechanism, MALAT1 decreases miR-124 availability, leading to enhanced epithelial-to-mesenchymal transition, cell migration, and invasion. Restoration of miR-124 expression was able to counteract the pro-tumorigenic effects of MALAT1 overexpression [67].

Additional support for lncRNA-mediated miRNA sequestration comes from studies on HOTAIR. In gallbladder cancer, HOTAIR was shown to directly bind miR-130a and associate with AGO2-containing RISC complexes, consistent with a ceRNA interaction.

HOTAIR and miR-130a were inversely expressed in tumor tissues. Reciprocal gain- and loss-of-function experiments demonstrated that HOTAIR functions as a molecular sponge, thereby reducing miR-130a availability and promoting tumor cell proliferation and invasion [68].

Multiple studies have reported that the long non-coding RNA H19 can regulate microRNA activity by acting as a competitive endogenous RNA (ceRNA).

In colorectal cancer, the action of H19 as a molecular sponge for miR-138 and miR-200a has been identified, thereby inducing a reduction in their inhibitory actions on target genes and thus stimulating epithelial–mesenchymal transition (EMT) and tumor progression. Luciferase reporter assays and gain- and loss-of-function experiments have indeed shown that overexpression of H19 enhances the migratory and invasive properties of colorectal cancer cells, while its silencing suppresses EMT-associated phenotypes [65].

Additional evidence supports the oncogenic role of lncRNA H19 as a competitive endogenous RNA (ceRNA) in clear cell renal cell carcinoma (ccRCC). In this context, H19 is upregulated and functions as a molecular sponge for miR-29a-3p, thereby attenuating its inhibitory effect on the downstream target E2F1. This H19/miR-29a-3p/E2F1 ceRNA regulatory axis contributes to the modulation of E2F1 expression and promotes ccRCC cell proliferation, suggesting a functional role in ccRCC pathogenesis. Collectively, these findings indicate that H19 may represent a potential therapeutic target in clear cell renal cell carcinoma [66].

In pancreatic cancer, the long non-coding RNA DANCR is upregulated in tumor tissues and promotes cancer cell proliferation, migration, and invasion by acting as a competitive endogenous RNA. DANCR acts as a molecular sponge, directly interacting with miR-33b, thereby reducing its miRNA-mediated effects, such as repression of downstream targets like MMP16. qRT-PCR analyses, luciferase reporter assays and functional inhibition experiments validated the involvement of lncRNA-miRNA DANCR/miR-33b regulatory network in pancreatic cancer progression [69].

Taken together, these studies demonstrate that lncRNA-mediated miRNA sponging constitutes a recurrent and functionally significant regulatory mechanism in cancer, promoting tumor progression through the reprogramming of miRNA-centered regulatory networks.

### 3.2. miRNA-Mediated Degradation or Silencing of lncRNAs

Although the traditional targets of miRNAs are protein-coding mRNAs, growing evidence shows that they can also bind directly to long non-coding RNAs (lncRNAs), leading to their degradation or silencing. From a mechanistic point of view, the interaction is based on sequence complementarity: miRNAs recruit the RNA-induced silencing complex (RISC) to target lncRNAs, resulting in post-transcriptional repression via degradation or translational inhibition. The RNA-induced silencing complex (RISC) is a ribonucleoprotein assembly in which a mature miRNA is incorporated into an Argonaute protein (AGO) [70].

The catalytic core of the RNA-induced silencing complex (RISC) is formed by the Argonaute proteins, which are responsible for miRNA-driven repression and destabilization/degradation of the target RNA, both coding and non-coding RNAs [71]. Guided by miRNAs, RISC thus recognizes complementary RNAs and induces their post-transcriptional silencing through mRNA degradation and/or translation repression. Recent studies have highlighted that miRNAs can directly regulate the stability of lncRNAs through RISC-mediated mechanisms [70,72].

In a pioneering study by Leucci E. et al., AGO2-dependent post-transcriptional silencing in cancer cells was shown to result from the direct interaction between miR-9 and the non-coding RNA MALAT1. Functional experiments showed that miR-9 overexpression significantly reduces MALAT1 levels, while AGO2 knockdown stabilizes MALAT1, confirming the involvement of the RISC machinery. Specifically, transcriptomic profiling (RNA-seq) revealed that miR-9 inhibition results in marked over-regulation of MALAT1, further confirming a direct regulatory effect. To further validate the specific binding between miR-9 and MALAT1, through canonical miRNA recognition elements, luciferase reporter assays with wild-type and mutated binding sites were used. Collectively, these results establish that miR-9 binds to MALAT1 in the nucleus in an AGO2-dependent manner, promoting its post-transcriptional degradation [73].

These results provide mechanistic evidence that miRNAs can act as direct silencers of lncRNAs, expanding the classical view of miRNA function beyond mRNA regulation.

### 3.3. lncRNA as Modulators of miRNA Biogenesis

Although the role of miRNAs as regulators of mRNA stability and translation is well established, it is only recently that their interaction with long noncoding RNAs has emerged. In recent years, several evidence have become recognized regarding the multiple mechanisms through which miRNA–lncRNA interactions operate. These include miRNA-mediated destabilization of lncRNA, lncRNA-mediated sequestration of lncRNA or competition for shared targets and lncRNAs that serve as miRNA precursors, collectively modeling the gene expression programs underlying major physiological and pathological processes. Beyond post-transcriptional regulation and ceRNA mechanisms, long non-coding RNAs have also been shown to modulate miRNA biogenesis at multiple levels. As described over the past decades, lncRNAs can interact with components of the miRNA-processing machinery, influencing pri- and pre-miRNA maturation and ultimately affecting miRNA availability. These observations highlight the multilayered regulatory potential of lncRNAs, adding an additional layer of control over gene expression beyond classical ceRNA networks [10,21,74].

In glioblastoma (GBM), the upregulated lncRNA MAFG-AS1 in tumor tissues has been shown to be a modulator of miR-34a biogenesis. Indeed, Zhao H et al. demonstrated through functional experiments that overexpression of MAFG AS1 reduces levels of mature miR-34a without affecting its precursor, indicating a specific interference with miRNA processing rather than transcription. Mechanistically, MAFG-AS1 affects components of the miRNA-processing machinery, probably including Drosha and Dicer, which are responsible for nuclear and cytoplasmic miRNA maturation, respectively. By reducing the maturation of miR-34a, MAFG-AS1 promotes the proliferation and migration of GBM cells, as confirmed by functional assays and in vivo xenograft models. Thus, an additional level of post-transcriptional control in cancer has been established by this study: lncRNAs can regulate miRNA maturation [75].

In line with the emerging roles of lncRNAs in miRNA biogenesis, Cahill HF. et al. demonstrated that the NRAD1 lncRNA, associated with triple-negative breast cancer (TNBC), influences mature miRNA levels and subcellular distribution, in part by modulating the miRNA-processing enzyme Dicer, highlighting a non-canonical mode of lncRNA-miRNA regulation in cancer. NRAD1 influences miRNA biology primarily through non-ceRNA mechanisms, including modulation of the miRNA biogenesis factor DICER, changes in miRNA subcellular localization, and reduced biogenesis of mitochondria-associated miRNAs such as miR-4485-3p, thereby contributing to cancer-associated gene expression changes [76].

Through these mechanisms, lncRNAs add an additional layer of regulation to miRNA-mediated gene silencing, expanding the complexity of non-coding RNA crosstalk in both physiological and pathological contexts.

### 3.4. Epigenetic Regulation Involving miRNA–lncRNA Crosstalk

Epigenetic regulation represents a key mechanism by which miRNA-lncRNA crosstalk modulates chromatin-modifying complexes, thereby shaping the transcriptional programs involved in cancer development and progression.

Polycomb-like proteins (PCLs) are essential accessory components of the PRC2.1 subcomplex. They mediate its recruitment to chromatin and promote gene repression through H3K27 trimethylation (H3K27me3), playing important roles in development and cancer [77]. Given the key role of PRC2 in regulating gene expression, its dysregulation in a wide range of tumors has been highlighted. In several solid tumors, including prostate cancer, bladder cancer, and melanoma, elevated PRC2 activity correlates with tumor-suppressor gene silencing, aggressive phenotypes, and advanced disease stages [78,79,80]. In contrast, in some cancers, including T-cell acute lymphoblastic leukemia (T-ALL) and breast cancer, PRC2 activity is reduced, thereby inducing increased oncogene expression [81,82].

Beyond their canonical role, PCLs interact with non-coding RNAs, which can guide or stabilize PRC2 at target genes or regulate the expression of Polycomb components themselves. In addition to chromatin-associated mechanisms, epitranscriptomic modifications of non-coding RNAs represent an additional regulatory layer in cancer. In particular, N6-methyladenosine (m6A) is a reversible RNA modification that regulates RNA stability and processing, thereby influencing gene expression programs in cancer. Dysregulation of the m6A machinery has been reported in several tumors, including hepatocellular carcinoma, glioblastoma, breast cancer, and lung cancer, where it has been implicated in cancer progression. Overall, m6A influences RNA regulatory networks in cancer [83].

Indeed, lncRNAs have been shown to associate with PRC2 and influence its recruitment to specific genomic loci [56], while microRNAs can modulate the expression of Polycomb proteins, such as EZH2, thereby establishing regulatory feedback loops.

In this context, Varambally S et al. demonstrated that loss of miR-101 in cancer leads to EZH2overexpression, the catalytic subunit of PRC2, providing direct evidence that microRNAs can directly regulate Polycomb components and thereby influence epigenetic gene silencing during tumorigenesis. Bioinformatics predictions and functional assays demonstrated that miR-101 directly targets the 3′ UTR of EZH2, thereby reducing the transcript and protein levels of EZH2 and other PRC2 components in tumor cell lines. Overexpression of miR-101 decreased global trimethylation of H3K27 and impaired cell proliferation, invasion and tumor growth in xenograft models, effects that could be rescued by expression of EZH2 lacking the 3’UTR. Furthermore, analysis of prostate cancer clinical samples revealed genomic loss of miR-101 loci correlates with elevated EZH2 expression, supporting a mechanistic role of miR-101 loss in epigenetic dysregulation during cancer progression [84].

Another key epigenetic mechanism involves histone deacetylases (HDACs), a group of enzymes involved in the removal of acetyl groups from histones and other proteins, promoting chromatin compaction and transcriptional repression. Dysregulation of HDACs, together with Polycomb-mediated silencing, may contribute to tumor development. Therefore, HDACs as an important therapeutic target are configured as an important therapeutic target and the possibility of using their inhibitors in the treatment of cancer opens up [85]. This is corroborated by the finding that HDAC4 suppresses the expression of miR-146a in esophageal cancer. HDAC4 silencing makes tumor cells more sensitive to radiation, raises miR-146a levels, and decreases the expression of its direct target IRAK1 [86]. When combined, these results imply that chromatin modifier and noncoding RNA dysregulation can alter gene expression by influencing the course of cancer and its response to treatment. Additionally, recent research further reveals that chromatin modifiers and non-coding RNAs are connected by intricate regulatory networks. HDACs may affect noncoding RNA circuits related to tumor progression and treatment response, consistent with HDAC6-driven LINC00152 upregulation in multiple myeloma, where it acts as a putative sponge for hsa-miR-499a-5p [87].

**Figure 1:**
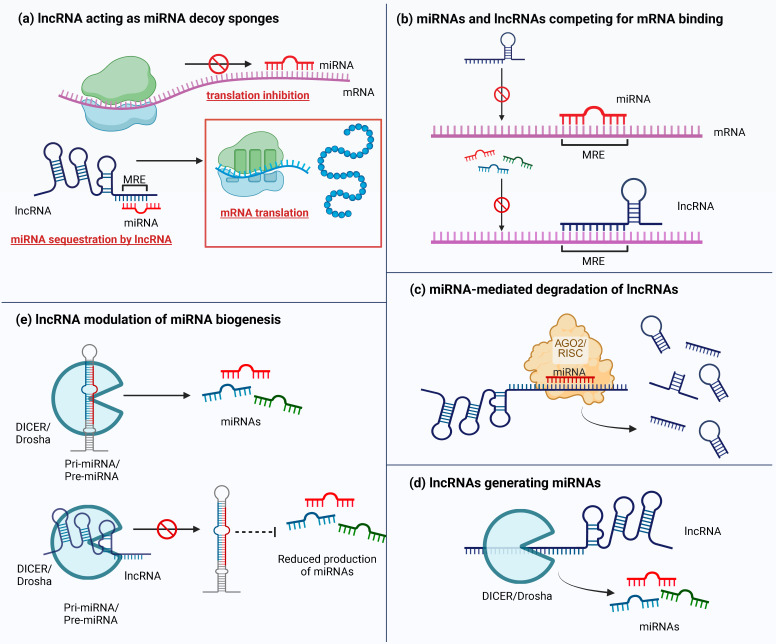
Molecular basis of miRNA–lncRNA cross-regulation: Overview of the main mechanisms involved in miRNA–lncRNA crosstalk in cancer. (**a**) lncRNAs can act as decoy sponges, sequestering miRNAs through shared MREs and reducing their availability for target mRNAs, thereby relieving post-transcriptional repression. (**b**) lncRNAs may also function as ceRNAs, competing with mRNAs for binding to the same MREs and influencing mRNA stability and translation. (**c**) In some cases, miRNAs directly bind to lncRNAs and promote their degradation through AGO2/RISC-dependent mechanisms. (**d**) Certain lncRNAs can act as precursors of miRNAs. (**e**) lncRNAs may also affect miRNA maturation by interfering with Drosha- or Dicer-mediated processing. Overall, these interactions highlight the complexity of miRNA–lncRNA crosstalk and its contribution to gene expression regulation in cancer. Created in BioRender. Scafuro, G. (2026) https://BioRender.com/nz88ak1.

Overall, these RNA-mediated mechanisms work alongside chromatin-modifying enzymes, such as PRC2 and HDACs, and coordinate both epigenetic and post-transcriptional regulation. Together, they influence gene expression programs critical for development, tumor response to treatment, stem cell maintenance, and cancer progression. The main mechanisms of miRNA–lncRNA cross-regulation are summarized in Figure 1.

## 4. miRNA–lncRNA Cross-Regulation in Key Hallmarks of Cancer Across Tumor Types

### 4.1. Integrated miRNA–lncRNA Networks in Cancer Hallmarks and Metastatic Progression

Beyond acting independently on mRNA targets, microRNAs (miRNAs) and long non-coding RNAs (lncRNAs) frequently engage in interconnected regulatory circuits that reshape cancer-associated gene expression programs. Through coordinated control of transcript stability, translation, chromatin-tethered transcriptional outputs, and intercellular communication, miRNA–lncRNA cross-regulatory networks may reprogram post-transcriptional regulation of signaling pathways controlling proliferation, survival, plasticity, genome stability, immune evasion, and stress responses. In this way, they promote cancer phenotypes in a range of tumor types [10].

Within this context of global tuning, the miRNA–lncRNA interface has become a key pillar of tumorigenic heterogeneity. These regulations extend beyond the canonical crosstalk of endogenous RNA sponging, giving rise to the control of miRNA turnover, inter- and intra-chromosomal interactions, and epigenetic reprogramming [88]. One structural element that may potentiate such effects is the existence of multiple miRNA response elements (MREs) in single lncRNAs, which would permit concurrent interactions with different miRNAs and increase the buffering capacity in ceRNA-like networks [89].

Competitive Endogenous RNA (ceRNA) regulation is among the most well-characterized interactions. In this case, lncRNAs act as sponges for miRNAs based on common MREs, decreasing their accessibility and consequently upregulating common mRNA targets [6,90]. LncRNAs may compete with mRNAs for miRNA binding, thus reducing miRNA-mediated repression and modulating characteristic phenotypes such as sustained proliferation capacity and resistance to cell death [89].

In addition, miRNAs can trigger lncRNA degradation via Ago2-dependent pathways and lncRNAs can conversely regulate miRNA expression at transcriptional or epigenetic level [10]. The biological significance of such interactions is very context-specific: transcript abundance, tumor microenvironment, and even dynamic stress conditions such as hypoxia and therapeutic pressure can modulate ceRNA stoichiometry and dictate whether an interaction can take place in vivo [91]. Although computational methods facilitate the identification of candidates, experimental confirmation is still necessary to establish their causal and phenotypic significance [92,93,94,95,96,97].

These ncRNA networks target multiple cancer hallmarks, such as uncontrolled proliferation, resistance to apoptosis and stress, epithelial-to-mesenchymal transition (EMT)-related plasticity and dissemination, angiogenesis, immune evasion, and metabolic reprogramming. Beyond classical endothelial-based angiogenesis, a subset of tumors also gain perfusion via vascular mimicry by generating microcirculatory-like arrangements without the need of endothelial cells. This mechanism represents an additional layer of vascular adaptation orchestrated by ncRNAs in aggressive tumors [98].

Several recurring hubs integrate these hallmark features, including the PI3K/AKT/mTOR axis, hypoxia–HIF pathways and epigenetic modifiers, such as EZH2. EZH2 circuits have been previously associated with the coordinated regulation of c-Myc and hypoxia signaling, providing a conceptual link between chromatin remodeling, angiogenesis, proliferation and metabolic adaptation [99,100]. In turn, HIF-1 signaling induces hypervascularization via upregulating growth factors, including VEGF and hepatocyte growth factor, thereby providing a mechanistic link between hypoxia-mediated metabolic stress and vascular remodeling [101].

The metastatic process, which drives the spread of malignant tumors to distant organs, is now known to involve multiple sequential, rate-limiting steps, including escape from the primary site, circulatory survival and transport, extravasation and colonization of distant tissues, where cancer cells are forced to adapt to new environmental constraints and to evade immune surveillance [102,103,104]. ncRNA networks can contribute to these adaptation processes by stabilizing survival programs and phenotypic plasticity in the context of selective pressure.

A cross-cutting theme linking ncRNA circuitry to metastasis is EMT/MET plasticity. EMT represents a dynamic and reversible program through which epithelial tumor cells acquire mesenchymal traits, whereas the reverse MET program supports efficient colonization during metastatic outgrowth [105,106]. Tumor cell plasticity enables cancer cells to disseminate from the primary tumor and enter the circulation, survive in the bloodstream, and eventually colonize distant tissues [107,108].

Core EMT transcription factors include Snail1/Snail2, Zeb1/Zeb2, and Twist1/Twist2, which are activated downstream of TGF-β, Wnt, Notch, and growth factor receptor signaling pathways [109,110,111,112,113,114,115]. lncRNAs can influence EMT-associated transcription by modulating chromatin structure and transcriptional activity. In parallel, microRNAs may directly affect the stability of lncRNA by triggering their decay. These regulatory interactions can generate feedback loops that help stabilize either epithelial or mesenchymal states [74,116,117].

In lung cancer, miR-217 suppresses EMT by limiting EZH2-dependent H3K27 trimethylation and by reducing MALAT1 expression through Ago2-mediated mechanisms [118].

Conversely, MALAT1 can promote EMT in triple-negative breast cancer by repressing miR-1 and thereby enhancing Slug-associated signaling pathways [119].

The lncRNA H19 provides another example of ncRNA-mediated EMT regulation. In colorectal cancer, H19 downregulates miR-138 and miR-200a, resulting in increased expression of the EMT drivers ZEB1 and ZEB2. A comparable mechanism has also been described in hepatocellular carcinoma, where H19 acts as a molecular sponge for let-7 and supports the maintenance of EMT-associated phenotypes [65,120].

Overall, these findings suggest that miRNA–lncRNA cross-regulation is embedded within broader regulatory networks that modulate EMT/MET plasticity, rather than representing isolated regulatory events [74]. Representative miRNA–lncRNA cross-regulatory axes across tumor types and associated cancer hallmarks are summarized in Table 1.

### 4.2. Cancer Types

#### 4.2.1. Breast Cancer

In breast cancer, interactions between miRNAs and lncRNAs contribute to several aspects of tumor progression, including proliferative signaling and cell-cycle control. These regulatory circuits can support cell proliferation by buffering tumor-suppressive miRNAs and maintaining cyclin/CDK-dependent transcriptional activity. The lncRNA HOTAIR has been shown to facilitate the G1/S transition through ceRNA-like regulatory activity by acting as a molecular sponge for miR-129-5p [121]. By contrast, reduction in MALAT1 expression has been reported to induce G1 arrest, suggesting an effect on checkpoint regulation [136].

Apoptosis resistance in breast cancer is likewise influenced by miRNA–lncRNA cross-regulatory networks. LncRNA-mediated sequestration of pro-apoptotic miRNAs can sustain survival signaling, as exemplified by UCA1, which has been reported to promote therapy tolerance through sponging of miR-18a targeting pro-survival pathways [122]. Autophagy frequently emerges as a stress-adaptive output under therapeutic pressure. ncRNA circuits converge on nodes such as Beclin1 and mTOR, and in triple-negative breast cancer a ceRNA-like axis involving lncRNA RMST and miR-4295 has been described to regulate ITPR1 expression in parallel with changes in proliferation, apoptosis, migration, and autophagy markers [123].

Dissemination and EMT plasticity are reinforced by reciprocal miRNA–lncRNA programs. MALAT1/miR-1 circuitry stabilizes EMT-associated transcriptional outputs, and metastatic traits can be transmitted intercellularly via extracellular vesicles, as illustrated by EV-mediated transfer of miR-200, promoting EMT and lung colonization in less metastatic recipient cells [119]. Angiogenic remodeling is likewise influenced by ncRNA regulation: miR-126 has been reported to restrain VEGF-A with downstream effects on apoptosis and vascular outputs [137], whereas exosomal circHIPK3 promotes endothelial angiogenesis by relieving miR-124-3p-mediated repression of MTDH [138]. Breast tumor progression occurs within a hypoxic and therapy-affected microenvironment, where ncRNA transfer through extracellular vesicles can influence both endothelial and immune cell compartments [139,140,141]. Some lncRNAs connect inflammatory and metabolic stress signaling to tumor progression. CamK-A has been found to activate NF-κB signaling, usually in response to inflammatory and metabolic stress, promoting a tumor-supportive microenvironment [142].

Overall, in breast cancer, miRNA–lncRNA interactions are particularly associated with epithelial plasticity, metastatic dissemination, and extracellular vesicle-mediated intercellular communication, reflecting the strong propensity of this tumor type for early dissemination and microenvironmental remodeling.

#### 4.2.2. Lung Cancer

miRNA-lncRNA interactions influence several aspects of lung cancer progression, including proliferation, survival signaling, epithelial–mesenchymal plasticity, and adaptation to the tumor microenvironment. One consequence of these regulatory circuits is disruption of checkpoint control and activation of CDK/E2F-driven transcription. The lncRNA TUG1 has been associated with cell-cycle progression and chemoresistance [143,144].

PI3K/AKT signaling is frequently involved in survival regulation in lung cancer. The lncRNA DLX6-AS1 has been reported to support lung cancer cell survival by sequestering miR-144 and promoting activation of PRR11 [126].

Regulatory circuits of ncRNAs can also reshape epithelial–mesenchymal plasticity and metastatic behaviors. Increased invasive and metastatic potential has also been linked to the MALAT1/miR-145-5p/NEDD9 axis [124].

Another aspect of ncRNA regulation involves control of RNA stability. MiR-217, for example, can trigger degradation of MALAT1, bringing to the reduction in EMT-associated transcriptional activity [125].

This interplay between tumor cells and neighboring tissues is also influenced by the contribution of angiogenesis, immune-related signaling and metabolic adaptation.

Several pro-angiogenic miRNAs, including miR-21-5p and miR-3157-3p, have been reported to promote vascular remodeling through repression of anti-angiogenic regulators and tumor-suppressor pathways [145,146].

The lung tumor microenvironment is frequently characterized by a hypoxic microenvironment. HIF-1α activation has been linked to increased PD-L1 expression and more invasive tumor behavior; inflammatory mediators such as IL-8 can further promote EMT and vascular remodeling [147,148].

In addition, lung tumors show extensive metabolic reprogramming. Hypoxia-driven HIF signaling influences angiogenesis, immune evasion and cellular responses to metabolic stress [149,150,151]. Overall, these observations support the idea that miRNA–lncRNA regulatory networks integrate tumor-intrinsic pathways with microenvironmental signals during lung cancer progression and are strongly shaped by hypoxia-driven signaling, inflammatory pathways, and metabolic adaptation characteristic of the pulmonary tumor microenvironment.

#### 4.2.3. Colorectal Cancer

In colorectal cancer, miRNA–lncRNA interactions participate in several mechanisms considered to be driving tumor progression, such as cell proliferation, resistance to apoptosis, EMT-associated dissemination, and remodeling of the tumor microenvironment. Some lncRNAs directly influence cell-cycle regulation. For instance, ROR1-AS1 has been reported to promote G1/S transition through EZH2-dependent repression of CDK inhibitors such as CDKN1A [152].

Survival signaling and therapy adaptation often involve activation of PI3K/AKT/mTOR pathways. In this context, tumor-suppressive lncRNAs such as MEG3 can affect therapy response, regulating apoptotic effectors in an miRNA-dependent manner [128].

EMT regulation in colorectal cancer is also influenced by lncRNA-mediated control of transcription factor networks. The lncRNA H19 promotes EMT by suppressing miR-138 and miR-200a, leading to increased expression of the EMT regulators ZEB1 and ZEB2 [65].

Communication through extracellular vesicles contributes to remodeling of the tumor microenvironment. Exosomal miR-25-3p promotes metastasis through targeting of KLF2 and KLF4, while exosomal miR-934 induces M2 macrophage polarization via PTEN/PI3K/AKT signaling [153,154].

Metabolic rewiring is another component of these regulatory networks. The lncRNA GLCC1 stabilizes c-Myc and enhances glycolytic activity, linking ncRNA-mediated regulation to tumor growth [155]. Overall, in colorectal cancer, miRNA–lncRNA interactions are closely linked to epithelial barrier disruption, inflammation-associated signaling, and microbiota-influenced tumor progression, highlighting the impact of the intestinal microenvironment on ncRNA regulatory dynamics.

#### 4.2.4. Prostate Cancer

In prostate cancer, miRNA–lncRNA interactions operate within the context of androgen receptor-driven transcriptional programs and contribute to the regulation of proliferation, apoptosis resistance, epithelial–mesenchymal transition (EMT), immune remodeling, and metabolic adaptation. Among the best-characterized examples, UCA1 promotes proliferative phenotypes through sequestration of miR-184, thereby sustaining oncogenic signaling outputs and tumor cell growth [129]. The androgen-regulated lncRNA PCGEM1 has similarly been associated with enhanced proliferation and colony formation, accompanied by RB phosphorylation and reduced p21 expression, consistent with reinforcement of cell-cycle progression programs [156,157].

In prostate cancer, the regulatory networks of ncRNAs contribute to cellular adaptation to stress and to therapeutic responses. Hypoxia-associated miRNAs, including miR-301a/b and miR-96, have been implicated in the regulation of autophagy and cell viability [158,159]. In a similar context, the lncRNA PCGEM1 has been linked to reduced apoptosis after chemotherapy, suggesting that ncRNA circuits can support tumor cell survival under treatment pressure [160].

Tumor invasiveness and progression are also modulated by specific miRNA-driven regulatory axes. For instance, miR-195 has been shown to target FGF2, thereby influencing tumor behavior [161]. In parallel, androgen receptor silencing has been associated with enhanced invasiveness through activation of the CCL2/CCR2–STAT3 signaling pathway [162].

Additional interactions between lncRNAs and miRNAs further contribute to these regulatory programs. PlncRNA-1 is an lncRNA that can protect androgen receptor transcripts from repression by miR-34c and miR-297, thereby promoting TGF-β1-related EMT signaling [130]. Overall, these observations indicate that ncRNA regulatory circuits in prostate cancer act as an androgen-associated layer of control linking transcriptional regulation, survival signaling, and adaptive tumor evolution, and are uniquely integrated with androgen receptor-driven transcriptional programs, thereby influencing hormonal responsiveness, therapy resistance, and androgen-dependent tumor progression.

#### 4.2.5. Hepatocellular Carcinoma

In hepatocellular carcinoma (HCC), miRNA-lncRNA interactions affect several processes associated with tumor progression, including proliferation, autophagy, epithelial–mesenchymal transition (EMT), angiogenesis, immune escape, and metabolic reprogramming. Many of these ncRNA circuits converge on key oncogenic signaling pathways that regulate tumor growth and adaptation.

Cell proliferation in HCC is strongly influenced by ncRNA regulatory circuits. Several lncRNAs affect CDK/E2F-dependent control of the cell cycle [163,164]. For example, Lnc-UCID promotes cell-cycle progression by increasing CDK6 expression through interaction with DHX9, whereas CASC11 stabilizes E2F1 mRNA via recruitment of EIF4A3 and supports transcriptional programs associated with uncontrolled proliferation [165,166].

The lncRNA MIAT clearly shows how ncRNA networks integrate different regulatory layers. Through interactions with miR-214 and miR-22-3p, MIAT modulates EZH2/β-catenin and SIRT1 signaling, influencing tumor-suppressive pathways such as p53/p21 and p16/pRb [167,168].

Cross-talk among ncRNAs also affects autophagy regulation in HCC. The lncRNA HOTAIR regulates the expression of ATG3 and ATG7 [169], while miR-541 targets RAB1B and ATG2A, influencing autophagic flux and responsiveness to sorafenib [170]. The lncRNA HULC has also been linked to protective autophagy under chemotherapeutic stress and also seems to support tumor cell survival during treatment [171].

lncRNA–miRNA interactions also contribute to EMT plasticity in HCC. LncRNA-ATB induces EMT by sponging members of the miR-200 family and activating ZEB-dependent transcriptional programs [131].

ncRNA interactions also influence angiogenic signaling in HCC. miR-126 targets VEGF, whereas MALAT1 modulates VEGF-A expression and contributes to pro-angiogenic activity within the tumor microenvironment [172,173]. These regulatory circuits are also involved in immune evasion. The lncRNA SNHG3 regulates PD-1 expression through miR-214-3p and may promote HCC recurrence by regulating immune infiltration [132].

Metabolic adaptation in HCC is also influenced by ncRNA signaling. Tumor-associated macrophage-derived exosomal lncMMPA promotes glycolytic reprogramming through upregulation of ALDH1A3 and supports tumor growth within the microenvironment [174]. ncRNA regulatory circuits also contribute to therapy resistance. Axes involving SNHG1 and NEAT1 interact with Akt- and c-Met-associated pathways and promote tumor cell survival under therapeutic pressure [175,176]. Many of these resistance mechanisms converge on common oncogenic signaling pathways, indicating that ncRNA networks reinforce pathway activity during treatment.

Overall, in HCC, ncRNA regulatory circuits are strongly interconnected with metabolic rewiring, chronic inflammation, and hypoxia adaptation, reflecting the unique metabolic and inflammatory environment of the liver.

#### 4.2.6. Head and Neck Squamous Cell Cancer (HNSCC)

In head and neck squamous cell carcinoma (HNSCC), interactions between miRNAs and lncRNAs contribute to several key aspects of tumor biology, including vascular remodeling, metabolic adaptation within the stroma, proliferative signaling, and invasive behavior. Rather than acting in isolation, these regulatory layers often operate through competing endogenous RNA mechanisms or miRNA-dependent transcriptional modulation, thereby connecting intracellular signaling with the surrounding tumor microenvironment.

One of the most evident outcomes of this cross-talk is the regulation of angiogenesis. For instance, the LINC00668/miR-297/VEGF-A axis has been shown to enhance angiogenic activity, ultimately supporting both vascular remodeling and tumor expansion [133]. Similarly, miR-130b-3p-mediated repression of MBNL1 has been associated with increased angiogenesis and more aggressive disease progression in HNSCC [177].

Beyond vascular regulation, ncRNA signaling also shapes metabolic interactions between tumor cells and stromal components. In oral squamous cell carcinoma, the H19/miR-675-5p/PFKFB3 axis promotes glycolytic reprogramming in cancer-associated fibroblasts, thereby enhancing their ability to support tumor growth [134].

Within tumor cells, multiple lncRNA–miRNA pathways sustain malignant phenotypes. For example, MALAT1 can suppress tumor-suppressive miRNAs such as miR-125b, leading to increased proliferation and invasion [178]. In parallel, HOTAIR-mediated inhibition of miR-7 contributes to the activation of oncogenic signaling pathways and further tumor expansion [179].

Importantly, these regulatory networks are not confined to cancer cells alone but also extend to vascular, stromal, and metabolic compartments of the tumor microenvironment. Processes such as angiogenesis, immune escape, and treatment resistance are highly interconnected in HNSCC, and miRNA–lncRNA interactions add an additional layer of regulation that integrates these elements within the broader framework of tumor progression [180]. Overall, in HNSCC, miRNA–lncRNA interactions are tightly connected to epithelial differentiation states and immune regulatory pathways, frequently in the context of environmental carcinogens or HPV-driven tumorigenesis, thereby contributing to tumor heterogeneity and adaptive progression.

#### 4.2.7. Leukemias and Lymphomas

In hematologic malignancies, such as acute and chronic leukemias and lymphoid neoplasms, ncRNA-mediated regulation is tightly embedded within lineage-specific transcriptional programs and differentiation states. Changes in the expression of leukemia-associated miRNAs, as well as ultraconserved genomic regions, have been consistently linked to disease phenotype, progression, and clinical outcome, highlighting the relevance of ncRNA signaling in hematopoietic transformation [181,182].

miRNA–lncRNA cross-regulation represents an additional level of control over leukemic cell behavior. Rather than acting through a single mechanism, these interactions can influence survival, proliferation, differentiation, and treatment response through a combination of ceRNA-like effects and more direct post-transcriptional regulation of key signaling pathways involved in disease progression [10,89].

A number of lncRNAs have been implicated in leukemia-associated miRNA networks. HOTAIR, for instance, has been linked to increased proliferation and survival, partly through its ability to modulate tumor-suppressive miRNAs and reinforce oncogenic signaling programs [183]. NEAT1 appears to act in a similar direction, attenuating miRNA-mediated repression and contributing to both enhanced proliferation and resistance to apoptosis [135]. UCA1 has also been associated with proliferative signaling and chemoresistance in acute myeloid leukemia, mainly through miRNA-dependent effects on PI3K/AKT-related survival pathways [184].

In parallel, miRNA-dependent stress-response pathways contribute to the treatment response of leukemic cells. In chronic myeloid leukemia, miR-21 has been shown to regulate autophagic flux by targeting core components such as Beclin-1, Vps34, and LC3-II, thereby linking ncRNA activity to cell survival under therapeutic pressure [185].

These regulatory effects are further influenced by signals coming from the immune system and the surrounding microenvironment. Inflammatory pathways, stromal interactions, and immune regulatory networks all intersect with ncRNA-mediated regulation, collectively supporting leukemic cell maintenance and disease evolution [186].

Overall, miRNA–lncRNA cross-talk adds an additional layer of complexity to leukemogenesis, integrating lineage-specific transcriptional programs with survival signaling, stress adaptation, and microenvironmental cues. Notably, in hematologic malignancies, these interactions are more tightly associated with differentiation states and hematopoietic hierarchy than with epithelial plasticity or angiogenesis, which are more characteristic of solid tumors. As in other cancer types, their functional impact remains highly context-dependent and is shaped by factors such as transcript abundance, cellular composition, and therapeutic pressure.

#### 4.2.8. Concluding Synthesis: Recurrent Hubs and Selective Pressures

Taken together, miRNA–lncRNA interactions can be viewed as a flexible regulatory layer that connects different RNA-based mechanisms, including ceRNA competition, miRNA-driven lncRNA decay, and feedback loops, with broader cancer phenotypes in both solid and hematologic tumors. Rather than acting through isolated pathways, these networks tend to converge on recurrent signaling hubs such as PI3K/AKT/mTOR, hypoxia–HIF pathways, and epigenetic regulators. At the same time, they are shaped by the tumor microenvironment and by selective pressures imposed by therapy, which together contribute to maintaining malignant traits while allowing a certain degree of plasticity and adaptation during disease progression [89,99,102]. Importantly, the functional impact of miRNA–lncRNA interactions is highly context-dependent and reflects the biological features of each cancer type, including hormonal regulation, hypoxia, metabolic specialization, and differentiation state.

## 5. Therapeutic Targeting of miRNA–lncRNA Networks

### 5.1. Biological Rationale for Targeting ncRNA Regulatory Networks

The capability of ncRNA to regulate oncogenes and tumor-suppressive pathways provides a strong biological rationale for their therapeutic targeting in cancer. Individual miRNAs often control multiple transcripts within interconnected signaling pathways, meaning that modulation of a single dysregulated miRNA can influence broader molecular networks rather than a single target [187,188,189]. This property underlies the development of miRNA-based therapeutic strategies. Such approaches aim either to restore tumor-suppressive miRNA activity or to inhibit oncogenic miRNAs through chemically engineered oligonucleotides [20,190,191,192,193]. Dysregulated miRNA expression has been reported across many tumor types. Experimental correction of miRNA levels can alter malignant phenotypes, supporting the development of RNA-based therapeutic approaches [194].

More broadly, ncRNAs operate within interconnected regulatory systems in which mRNAs, miRNAs and lncRNAs collectively determine transcript stability and translational output. Competitive RNA interactions can dynamically redistribute regulatory pressure among transcripts, meaning that modulation of a single ncRNA may propagate effects across entire circuits [2,6,195]. Suppression of oncogenic lncRNAs may therefore release tumor-suppressive miRNAs previously sequestered by competitive binding and restore repression of downstream oncogenic targets [196]. Functional miRNA–lncRNA interactions include sponging, lncRNA-derived miRNA production, miRNA-directed lncRNA degradation, and competition for shared targets, collectively influencing tumor proliferation, apoptosis, metastasis and microenvironmental signaling [197,198,199,200,201,202,203,204,205]. Consequently, targeting dysregulated ncRNA circuits has emerged as a strategy to limit tumor progression and therapy resistance [197,206].

### 5.2. Therapeutic Modulation of miRNAs

#### 5.2.1. miRNA Inhibition Strategies

Therapeutic modulation of miRNAs is generally based on two complementary approaches: restoration of tumor-suppressive miRNAs through synthetic double-stranded mimics or vector-mediated expression, and inhibition of oncogenic miRNAs using chemically modified antisense oligonucleotides [20,207,208,209,210,211].

Inhibitory strategies include ASOs, anti-miRs, LNA-modified oligonucleotides, and antagomirs, which bind to mature miRNAs and block their interaction with target transcripts [207]. These antisense molecules function through sequence complementary binding to the mature miRNA, thereby preventing regulatory activity and promoting functional silencing [186]. Efficient in vivo inhibition typically requires chemical optimization to enhance affinity, metabolic stability and pharmacokinetics, commonly using 2′-O-methyl, 2′-MOE, 2′-fluoro or locked nucleic acid modifications, with LNA chemistries providing particularly strong RNA binding [185,186,212,213,214,215,216,217,218,219].

Antagomirs—cholesterol-conjugated RNAs containing 2′-O-methyl and phosphorothioate modifications—were among the first reagents shown to enable efficient in vivo miRNA silencing [220,221,222]. Several alternative approaches have been proposed to modulate miRNA activity. These include miRNA masks designed to bind specific 3′UTR sites, as well as miRNA sponges that contain multiple binding motifs and competitively sequester endogenous miRNAs [223,224,225,226,227].

#### 5.2.2. miRNA Replacement Strategies

Replacement strategies are designed to restore tumor-suppressive miRNA activity using synthetic duplex mimics or vector-based expression systems [228,229,230,231].

These mimics typically contain a guide strand corresponding to the endogenous miRNA, which is preferentially incorporated into the RISC. The passenger strand is destabilized or chemically modified to promote guide-strand selection and may include delivery-enhancing conjugates such as cholesterol [189,207,232,233].

Chemical modifications of the ribose backbone, including 2′-fluoro substitutions, further increase resistance to degradation and improve intracellular stability [232].

Despite these advances, duplex mimics can activate innate immune-sensing pathways and may distribute to non-target tissues. For this reason, physiological dosing and tissue-specific delivery remain important challenges for therapeutic application [234,235,236,237].

### 5.3. Chemical Optimization of RNA Therapeutics

The clinical feasibility of miRNA-based therapeutic approaches depends, to a large extent, on the chemical design of the oligonucleotides. A major limitation is their rapid degradation by nucleases. This issue can be mitigated by introducing phosphorothioate (PS) backbone modifications, which consist of replacing the non-bridging oxygen atoms with sulfur, a change that also influences the pharmacokinetic behavior of these molecules [222,238].

Improved stabilization can also be achieved through modifications of the ribose sugar. In particular, the replacement of the 2′-OH group increases resistance to intracellular nucleases and improves molecular stability. Several ribose modifications are commonly used, including 2′-O-methyl (2′-OMe), which provides relatively simple and cost-effective stabilization; 2′-fluoro (2′-F), which enhances target-binding affinity; and 2′-O-methoxyethyl (2′-MOE), a bulkier modification that increases intracellular persistence [239].

Among these chemistries, a particularly distinctive class of modifications is represented by Locked Nucleic Acids (LNAs). By using a methylene bridge to ‘lock’ the ribose, they achieve an exceptionally high binding affinity, thereby enabling ‘tiny-LNA’ strategies that target the miRNA seed region [240]. Ultimately, combining LNAs with PS backbones provides the kind of metabolic stability that is non-negotiable for long-term clinical use [241].

### 5.4. Therapeutic Targeting of lncRNAs

Compared with miRNAs, lncRNA targeting remains less advanced, partly due to incomplete mechanistic characterization, although their tissue specificity and locus-dependent regulatory functions make them attractive intervention points [242]. RNA interference approaches using siRNAs can effectively suppress lncRNAs, including nuclear transcripts [243,244,245,246].

RNA interference is a sequence-specific post-transcriptional silencing mechanism triggered by small double-stranded RNAs of approximately 21 nucleotides that guide RISC-mediated cleavage of complementary transcripts [247,248,249,250,251,252,253,254]. Engineered siRNAs bind complementarily to mRNAs and promote degradation of the encoded transcript, typically with higher target selectivity than miRNA modulation [255,256,257].

Synthetic siRNAs generally produce transient gene silencing, while shRNAs expressed from viral or plasmid vectors can lead to a more sustained knockdown, depending on promoter activity and expression levels [258,259,260].

However, immune activation remains a significant limitation of RNA interference-based strategies. Long double-stranded RNAs are known to stimulate PKR and interferon responses, and siRNAs can also be detected by endosomal Toll-like receptors. Another concern is that very high levels of shRNA expression may saturate exportin-5, which can interfere with the normal processing of endogenous miRNAs [261,262,263,264,265].

Efficient in vivo delivery is also a major challenge. Negatively charged siRNA duplexes do not readily cross cellular membranes without delivery systems. Liposomes, nanoparticles, and ligand-conjugated platforms can protect RNA from degradation and facilitate receptor-mediated uptake. Chemical stabilization strategies further improve resistance to nuclease-mediated degradation [266,267,268,269,270].

Ribose modifications increase stability, although incorrect placement may reduce Argonaute loading efficiency [271,272,273]. Cholesterol conjugation has also enabled systemic delivery with measurable in vivo knockdown [274].

Seed-dependent off-target repression can occur when partial complementarity exists within 3′UTRs, although predictive design tools and chemical optimization substantially reduce clinically relevant toxicity [275,276,277].

ASOs may alternatively induce RNase-H-mediated degradation of lncRNAs [278,279], while CRISPR–Cas systems enable programmable repression, activation or deletion of lncRNA loci, although off-target cleavage and delivery constraints still limit clinical translation [58,280,281,282,283].

### 5.5. Delivery Systems for ncRNA Therapeutics

**Figure 2:**
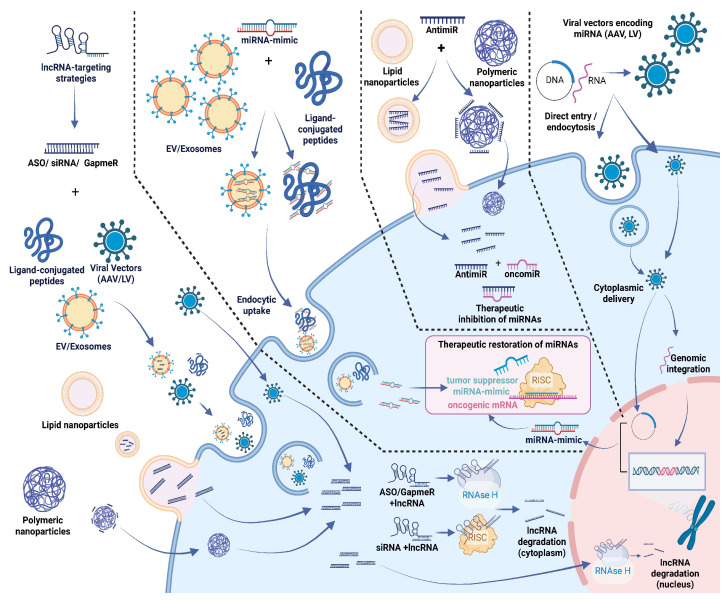
Therapeutic targeting of miRNA–lncRNA networks and delivery strategies in cancer. Schematic overview of current therapeutic strategies targeting oncogenic non-coding RNA (ncRNA) networks in cancer. AntimiRs and ASOs are used to inhibit oncogenic miRNAs, whereas miRNA mimics and viral vectors aim to restore tumor-suppressive miRNA activity. lncRNAs can be targeted through GapmeRs or siRNAs, promoting RNase H– or RISC-mediated degradation depending on subcellular localization. The figure also summarizes major delivery platforms, including lipid nanoparticles and extracellular vesicles/exosomes, and highlights key barriers to effective intracellular delivery, such as cellular uptake, endosomal escape, and tissue-specific targeting. Created in BioRender. Scafuro, G. (2026) https://BioRender.com/fts9ocr.

Efficient and safe intracellular delivery remains the principal bottleneck for RNA therapeutics. Systemically administered RNA molecules must overcome extracellular degradation, vascular barriers, uptake limitations, endosomal trapping and immune sensing [284,285,286,287]. An overview of therapeutic strategies and delivery approaches for ncRNA targeting is shown in Figure 2. Nanoparticle encapsulation—including lipid, polymer or emulsion systems—enhances systemic stability and tumor delivery efficiency [288]. Viral vectors can provide sustained expression but remain limited by immunogenicity and production complexity [289,290].

This figure captures the current strategies for dismantling oncogenic ncRNA circuits, where antimiRs and ASOs are used to sequester overexpressed miRNAs while synthetic mimics or viral vectors attempt to replenish lost tumor-suppressive functions. Therapeutic targeting of lncRNAs relies on approaches that are, in part, different from those used for miRNAs. Common strategies include GapmeRs and siRNAs, which promote RNA degradation through RNase H– or RISC-dependent mechanisms, depending on whether the target is mainly localized in the nucleus or in the cytoplasm. The figure also highlights some of the main challenges related to therapeutic delivery. Several systems have been explored, including lipid nanoparticles and extracellular vesicles, but efficient delivery remains a critical issue. For these approaches to be effective, RNA molecules need to enter cells, escape endosomal compartments, and reach the appropriate intracellular location. Further improvements in delivery platforms, together with more refined targeting strategies, will likely be required to overcome key translational limitations, particularly with regard to tissue specificity and off-target effects, and to enable effective modulation of ncRNA networks in cancer.

Non-viral lipid nanoparticles and liposomes protect RNA cargo, extend circulation time, and influence intracellular trafficking and endosomal escape [285,291,292]. Functionalization with targeting ligands can further improve tumor specificity. Extracellular vesicles and exosomes are also emerging as biologically compatible delivery carriers with potentially reduced immunogenicity and improved tissue penetration [293,294,295].

### 5.6. Clinical Translation and Current Limitations

Despite strong mechanistic rationale and extensive preclinical evidence, clinical translation of ncRNA therapeutics remains limited. Only a small number of miRNA-based agents have entered early-phase trials [296,297,298]. Additional representative clinical programs are summarized in Table 2. The miR-34a mimic MRX34 showed pharmacodynamic activity but was discontinued due to immune toxicity, while Miravirsen provided proof-of-concept for systemic anti-miRNA therapy despite resistance mutations [275,299,300,301].

This table summarizes key clinical-stage programs targeting non-coding RNAs. It highlights microRNA-based therapeutic strategies, including miRNA replacement and inhibition, together with saRNA/RNAa approaches and the use of lncRNA regulatory elements (e.g., the H19 promoter) to drive gene therapy. For contextual comparison, several landmark mRNA-targeting platforms (RNAi/ASO) are also included. Although these strategies target different RNA species, they face many of the same translational challenges as ncRNA-based therapies, including systemic delivery, endosomal escape, and unintended immune activation.

Major translational barriers include delivery inefficiency, limited tissue specificity, off-target interactions and context-dependent RNA function [293,307]. Additional constraints include nuclease degradation, endosomal escape limitations and restricted disease-site targeting [20,185,193,232]. Because miRNAs act pleiotropically, modulation of a single miRNA can generate transcriptome-wide effects [308,309,310]. Chemically modified oligonucleotides may also trigger complement activation, coagulation abnormalities or hepatotoxicity [311,312]. Determining dosing strategies that balance efficacy with immune safety therefore remains a central challenge [297].

Overall, therapeutic targeting of miRNA–lncRNA networks represents a compelling strategy for precision oncology, but its clinical success will depend on improved delivery technologies, refined network-level target selection and better control of immune and off-target toxicities.

## 6. Diagnostic and Prognostic Applications

The search for novel non-invasive and cost-effective biomarkers has received increasing attention in biomedical research [313]. In the last few years, an increasing number of scientific publications have reported the potential use of microRNAs (miRNAs) and long non-coding RNAs (lncRNAs) for diagnostic and prognostic purposes [314,315,316]. Currently, the majority of ncRNAs considered as liquid biopsy biomarker candidates are investigated mainly for diagnostic and screening applications [317,318]. Despite these promising findings, the sensitivity and specificity of these platforms remain critical issues that need to be addressed.

Among non-coding RNAs, miRNAs have attracted particular attention because of their abundance and intrinsic stability in a variety of bodily fluids (including blood, urine, stool, and saliva), making them attractive candidates for the development of non-invasive biomarkers [318,319,320]. In this context, the advent of nanotechnology has enabled the development of nanostructured biosensors, which are increasingly exploited in the diagnostic field for the sensitive and specific detection of circulating miRNAs [321,322]. Circulating miRNAs are able to reflect molecular alterations occurring within tumor tissues and may therefore provide useful information for cancer diagnosis and disease monitoring [323,324]. In parallel, emerging evidence indicates that lncRNAs can also be detected as cell-free RNAs in circulation and represent a promising additional source of non-invasive biomarkers [325]. The potential value as diagnostic biomarkers of several cancer-associated lncRNAs, such as UCA1, MALAT1 and H19, is supported by the fact that they have been reported to be significantly overexpressed in serum or plasma samples from cancer patients [326,327,328,329].

Since many ncRNAs are frequently overexpressed in tumors, they may also be useful for monitoring cancer progression, recurrence and response to treatment [330,331]. For this reason, circulating ncRNAs could potentially be used as companion diagnostic biomarkers in future ncRNA-based therapeutic trials.

More recently, the combination of miRNAs and lncRNAs has been proposed as an advanced biosignature strategy. Integration of these RNA molecules may help overcome the sensitivity and specificity limitations typically associated with single biomarkers. Indeed, several studies have shown that combined miRNA–lncRNA signatures achieve higher diagnostic accuracy and improved prognostic performance compared with individual miRNAs or lncRNAs alone, as they capture complementary layers of post-transcriptional regulation [6,198,329,332]. Therefore, the combined analysis of these molecules may provide a more accurate representation of tumor biology.

These advanced biosignatures may have broad applications in oncology. For example, they could help distinguish between healthy and tumor tissues, stratify tumor subtypes, and monitor therapeutic responses. Altered expression of ncRNAs has been widely linked to cancer initiation and progression, which has driven growing interest in their use as diagnostic and prognostic biomarkers [333,334,335].

However, the relevance of combined miRNA–lncRNA signatures is not limited to oncology. Similar patterns have also been reported outside of cancer. In cardiovascular disease, for instance, changes in ncRNA networks have been linked to myocardial injury and to the processes driving cardiac remodeling. In metabolic conditions such as type 2 diabetes, these RNA signatures appear to reflect underlying alterations like β-cell dysfunction, insulin resistance, and chronic inflammation.

Something comparable has been observed in autoimmune and inflammatory diseases, including rheumatoid arthritis and inflammatory bowel disease, as well as in renal and pulmonary disorders. In these settings, RNA-based signatures often mirror ongoing tissue damage and fibrotic changes, and in some cases, they seem to track with treatment response.

Chronic inflammatory diseases are a good example of this. In ulcerative colitis, dysregulated ncRNAs have been associated with defects in the epithelial barrier, immune imbalance, and persistent mucosal inflammation. Here, combined miRNA–lncRNA signatures—whether detected in tissue or in circulation—may provide information on disease activity, response to therapy, or even the risk of progression to colorectal cancer.

Along the same lines, several studies have pointed to the prognostic value of integrated non-coding RNA signatures across different tumor types.

A systematic analysis of an lncRNA–miRNA–mRNA competing endogenous RNA (ceRNA) network in breast cancer identified a four-lncRNA signature capable of stratifying patients into distinct risk groups. Notably, this combinatorial model outperformed single RNA-based biomarkers in risk stratification and showed a stronger association with overall survival, highlighting the added prognostic value of integrated ncRNA networks [320]. Similarly, an integrated miRNA–lncRNA expression signature in ovarian cancer has been shown to predict survival outcomes in patients with wild-type BRCA1/2, highlighting the potential relevance of ncRNA interactions beyond conventional genetic markers [336]. Moreover, integrated analyses of lncRNA, miRNA and mRNA expression profiles have been used to develop prognostic models. These integrated models significantly improved survival prediction compared not only with traditional clinicopathological parameters but also with single-layer RNA biomarkers [337].

Additional evidence also supports the clinical value of combining miRNAs and lncRNAs into prognostic signatures. In breast cancer, for example, the combined evaluation of the lncRNA H19 and the miRNA let-7a has been shown to predict patient survival more accurately than either biomarker alone, demonstrating improved prognostic power when these molecules are analyzed in combination rather than individually [338,339]. In triple-negative breast cancer, plasma levels of lncRNA NEF and miR-155 showed opposite expression patterns in patients compared with healthy individuals. Importantly, their combined assessment provided a more robust association with clinical outcome than either marker alone, supporting their use as integrated diagnostic and prognostic biomarkers [340].

Collectively, these findings indicate that combinatorial miRNA–lncRNA signatures consistently provide superior diagnostic and prognostic performance compared with individual ncRNA biomarkers, supporting their potential clinical utility as more reliable and informative tools for disease detection and patient stratification. However, despite these promising results, standardized analytical workflows, large multicenter validation studies, and well-designed clinical trials will be required before these approaches can be translated into routine clinical practice.

## 7. Computational and Systems Biology Approaches

### 7.1. Database for Predicting miRNA-lncRNA Interactions

Interactions between microRNAs (miRNAs) and long non-coding RNAs (lncRNAs) play an important role in post-transcriptional gene regulation and have been widely studied in the context of competing endogenous RNA (ceRNA) [6] networks. lncRNAs can function as ceRNAs, molecular sponges, or regulators of miRNA biogenesis. To characterize these interactions, several bioinformatics databases have been developed which integrate sequence complementarity, thermodynamic stability, evolutionary conservation, expression correlation and high throughput experimental evidence. There are many databases which can be used to predict miRNA-lncRNA interactions, some of them are DIANA-lncBase, starBase, miRcode, LncRNASNP, NPInter and RNAInter.

One of the commonly used bioinformatics tools is DIANA-LncBase [341], which provides both experimentally validated and computationally predicted miRNA-lncRNA interactions. LncBase integrated prediction algorithms such as miRanda and TargetScan-derived features and incorporates Argonaute-crosslinking and immunoprecipitation sequencing (AGO-CLIP-Seq) datasets to enhance confidence scoring. The clear separation between predicted and experimentally supported interactions makes it suitable for in silico screening and downstream network analysis.

starBase is a CLIP-Seq-driven database [15] that infers miRNA-lncRNA interactions by integrating large-scale High-Throughput sequencing of RNA isolated by Crosslinking and Immunoprecipitation (HITS-CLIP), Photoactivatable-Ribonucleoside-Enhanced Crosslinking and Immunoprecipitation (PAR-CLIP), Individual-nucleotide resolution UV Crosslinking and Immunoprecipitation (iCLIP) and Cross-linking, Ligation, and Sequencing of Hybrids (CLASH) datasets. From a bioinformatics perspective, starBase is especially valuable because it reduces false-positive predictions by anchoring interactions to experimentally derived RNA–protein binding sites, enabling robust reconstruction of ceRNA networks.

miRcode is a commonly used tool for predicting miRNA response elements within long non-coding transcripts based on GENCODE annotations [94]. The database mainly relies on sequence-based prediction and does not provide experimental validation. It is frequently employed for hypothesis generation and for the initial identification of potential miRNA-lncRNA interactions.

Other resources, including LncRNASNP, NPInter and RNAInter, broaden the analysis of ncRNA interactions by incorporating additional types of information such as genetic variants, RNA–protein interactions, and datasets derived from different species. Among these, RNAInter functions as an integrative repository that collects both predicted and experimentally supported RNA-associated interactions within a unified annotation system.

Because databases differ in prediction algorithms, annotation versions, and confidence criteria, bioinformatic studies often compare results across multiple resources. In practice, consensus-based filtering strategies are commonly applied in order to increase the reliability of predicted interactions (Table 3).

### 7.2. Big Data, Multi-Omics Integration and Network Biology

#### 7.2.1. Big Data

Big data refers to enormously large, complex and heterogeneous datasets that surpass the capabilities of traditional data management and analytical tools. Analysis of miRNA-lncRNAs interactions involve the integration of large-scale datasets generated by high-throughput technologies [344]. Genome-wide transcriptomic profiling approaches, such as RNA sequencing, generate large datasets that capture the expression dynamics of both miRNAs and lncRNAs across multiple biological conditions and disease states [345]. The combinatory nature of miRNA-lncRNA interactions, where a single miRNA can target multiple lncRNAs and vice versa, further amplifies data complexity and necessitates big data analytical frameworks [6]. Big data analysis of miRNA-lncRNA interactions provided many insights into tumor initiation, progression and its therapeutic resistance [346]. Dysregulated lncRNAs can alter miRNA availability through competing endogenous RNA (ceRNA) mechanisms, which can help in reshaping gene expression networks and altering oncogenic signaling pathways [347]. Integration of big data such as RNA-seq, miRNA-seq and other clinical datasets have enabled the identification of prognostic miRNA-lncRNA interaction signatures and potential therapeutic targets [348]. Yet, challenges related to data normalization, batch effects and reproducibility remain major obstacles to translating big data-derived miRNA-lncRNA interactions into clinical applications [344].

#### 7.2.2. Multi-Omics Integration

Multi-omics approaches combine large datasets generated across many molecular layers, including genomics, proteomics, transcriptomics, epigenomics, metagenomics and other clinical datasets [349,350]. In non-coding RNA research, big data enables the integration of miRNA and lncRNA expression with mRNA profiles and epigenetic data, thereby uncovering multi-layered regulatory interactions that single-omics approaches fail to resolve [351]. The scale, heterogeneity and high dimensionality of these datasets require advanced data integration strategies, like network inference, matrix factorization and ML models which are the hallmarks of big data analytics [352].

To prioritize biologically relevant miRNA–lncRNA interactions, it is necessary to consider them within the broader regulatory context of the cell. Integrating multi-omics datasets allows these interactions to be interpreted within complex regulatory networks rather than as isolated molecular events [353]. Large-scale analyses of public datasets have also highlighted that these interactions are highly context-dependent. Their activity is influenced by factors such as chromatin organization and transcriptional dynamics, emphasizing the need for integrative and data-driven systems-level approaches to better understand ncRNA regulatory networks [354].

Interpreting these datasets remains challenging. High-throughput analyses are often affected by issues such as data sparsity, batch effects, and the still incomplete annotation of many lncRNAs. Despite these limitations, large-scale data integration remains essential for identifying robust regulatory relationships and extracting biologically meaningful signals from complex datasets relevant to disease mechanisms [350].

#### 7.2.3. Network Biology

High-throughput technologies generate vast amounts of data, but translating this information into biological insight is not always straightforward, especially without a broader, system-level view. Network biology offers a useful way to approach this complexity by organizing large datasets into interconnected frameworks [353,355]. In this context, biological entities—such as genes, proteins, miRNAs, and lncRNAs—are treated as nodes, while their interactions, whether regulatory or physical, are represented as edges.

Looking at the data this way helps move beyond simple lists of molecules. Instead, it allows different types of omics data to be integrated within the same framework, making it easier to identify functional modules and key regulatory hubs that might otherwise go unnoticed when each component is analyzed in isolation [356].

In the context of ncRNA regulation, network biology has become crucial for characterizing miRNA-lncRNA interaction networks and their roles in gene regulatory circuits [2]. By integrating transcriptomic, epigenomic and interaction data, network models allow the systematic reconstruction of regulatory and competing endogenous RNA (ceRNA) networks and the identification of hub miRNAs or lncRNAs with central roles in disease-associated pathways [6,357].

Network-based analyses applied to large-scale datasets, such as cancer multi-omics, which provides insights into disease mechanisms, and therapeutic vulnerabilities, highlighting the value of network biology in translating big data into biological knowledge [353].

## 8. Challenges and Future Perspectives

Despite the progress made in understanding ncRNA biology, translating these findings into clinical applications remains challenging. Evidence on miRNA–lncRNA cross-regulation is still growing, and these networks appear to be involved in multiple aspects of cancer, including diagnosis, prognosis, and therapeutic response. At the same time, several conceptual and practical issues remain unresolved. In particular, the functional relevance of ceRNA-mediated interactions is still debated, as it depends on strict quantitative and stoichiometric constraints. A detectable ceRNA effect emerges only when the total abundance of available competing binding sites becomes comparable to or exceeds the intracellular miRNA pool, indicating a clear stoichiometric threshold [358,359]. Coherently, miRNAs are often present in excess relative to competing transcripts, including lncRNAs, thereby substantially limiting the likelihood of effective functional sequestration [360]. Moreover, the literature has shown that many reported effects arise from non-physiological overexpression systems that do not reflect endogenous cellular conditions [361], while miRNA-target competition is generally limited even in global quantitative analyses [362]. Overall, the evidence supports a highly context-dependent and quantitatively constrained role of ceRNA interactions, rather than a general regulatory mechanism. Addressing these quantitative and methodological limitations will be essential for clarifying the biological and clinical relevance of ceRNA networks and to support their potential translational applications.

### 8.1. Technical Limitations and Experimental Validation Challenges

A key unresolved issue concerns the validation of predicted miRNA–lncRNA interactions. Computational tools such as DIANA-LncBase, starBase, and miRcode are valuable for generating candidate interactions, but they often produce extensive lists based largely on sequence complementarity. Translating these predictions into experimental evidence is not straightforward. Validation requires time, optimization, and considerable resources, making it difficult to systematically assess all predicted interactions in most experimental settings.

Additional complexity arises from the organization of RNA regulatory networks. A single miRNA can target multiple lncRNAs, while individual lncRNAs may interact simultaneously with several miRNAs. This many-to-many architecture complicates both computational prediction and experimental validation, and it is not always clear which interactions are functionally relevant.

Furthermore, not all detected transcripts are necessarily biologically active. In particular, many lncRNAs remain poorly characterized, and distinguishing functional molecules from transcriptional background remains challenging. Within ceRNA networks, regulatory effects also depend on specific stoichiometric relationships between interacting molecules, further limiting the subset of interactions that are likely to be biologically meaningful.

Quantitative studies suggest that these interactions are likely to occur only under specific abundance conditions, which may limit their impact in vivo [358,363]. As a result, only a subset of the predicted interactions is expected to be biologically meaningful.

From a methodological standpoint, commonly used approaches also come with their own limitations. RNA interference- or antisense-based strategies can affect both RNA-mediated functions and transcriptional output, making the results harder to interpret than expected. Similarly, CRISPR-based perturbation of lncRNA loci does not always produce clear loss-of-function phenotypes, likely because of the structural and regulatory complexity of these regions [364]. For this reason, combining different strategies is often necessary, including crosslinking-based approaches such as CLIP, to obtain more reliable evidence of direct interactions [365,366].

### 8.2. Tumor Heterogeneity and Context Dependency

Tumor heterogeneity represents an additional source of complexity. Variability between patients is well recognized, but significant differences can also be observed within the same tumor, with clear consequences for ncRNA expression patterns.

Tumors are composed of multiple cell populations, including malignant cells, stromal elements, immune infiltrates, and cancer-associated fibroblasts, each contributing differently to the overall transcriptional landscape. As a result, interactions inferred from bulk transcriptomic data may not fully capture the regulatory dynamics occurring at the cellular level. This may partly explain the variability observed in the performance of some ncRNA-based biomarkers across different studies.

Insights from single-cell approaches have further emphasized this point, showing that distinct cell populations within the same tumor can display marked differences in both transcriptional profiles and genomic alterations [367,368]. Although this heterogeneity complicates the identification of broadly applicable regulatory patterns, it also highlights the potential of ncRNAs as cell-type-specific markers, particularly in the context of precision medicine.

### 8.3. Emerging Single-Cell and Spatial Transcriptomics Approaches

To address some of these limitations, there has been increasing use of single-cell RNA sequencing and spatial transcriptomics. Compared with bulk approaches, these methods allow a more refined analysis of ncRNA expression and of the regulatory interactions occurring within tissues.

Single-cell RNA sequencing enables the identification of miRNA–lncRNA interactions at the level of specific cell populations, which is particularly relevant in heterogeneous tumor contexts. Spatial transcriptomics provides complementary information by preserving tissue architecture and allowing RNA expression to be interpreted within its native spatial context [369,370]. This is especially useful for examining the relationships between tumor cells, stromal compartments, and immune infiltrates. Beyond their descriptive capabilities, these approaches are increasingly integrated with multi-omics and computational frameworks to reconstruct cell-type-specific regulatory networks. In particular, the combination of single-cell transcriptomics with small RNA profiling and advanced network inference methods enables the identification of functional miRNA-lncRNA interactions within defined cellular populations [371,372]. These strategies allow the dissection of dynamic and context-dependent regulatory programs, revealing ncRNA interactions associated with specific cellular states such as epithelial-to-mesenchymal transition, stemness, and therapy resistance [373]. Furthermore, the integration of spatial and single-cell data provides insights into intercellular communication networks, including those between tumor and microenvironmental components [374]. From a translational perspective, these high-resolution approaches may facilitate the identification of cell-type-specific ncRNA signatures with diagnostic and prognostic potential, as well as discovery of novel therapeutic targets within defined tumor niches [375,376].

However, these approaches still present some limitations. Current single-cell platforms are largely biased toward polyadenylated transcripts, meaning that a proportion of ncRNAs may not be captured [377]. Spatial methods can also be affected by issues such as limited resolution and signal diffusion, which may complicate data interpretation [378]. In addition, the analysis of these datasets remains challenging due to their complexity and high dimensionality.

Despite these constraints, ongoing technological improvements, including advances in single-cell multi-omics and enhanced detection of non-polyadenylated transcripts, are expected to enhance the reliability and applicability of these approaches. This will likely facilitate more accurate and context-specific characterization of ncRNA regulatory networks.

### 8.4. Challenges and Future Directions for RNA-Based Therapeutics

From a translational perspective, targeting miRNA–lncRNA networks sounds promising, but in practice it is still quite challenging. Nucleic acid-based therapeutics have some well-known limitations: they are not particularly stable, they can trigger immune responses, and getting them inside cells is not trivial. In the end, a lot depends on delivery—how well the molecules are protected, how long they remain stable in circulation, and whether they actually reach the right intracellular compartment.

Right now, delivery is still one of the main bottlenecks [379]. Toxicity is another aspect that cannot be ignored, especially immune-related effects. Formulation is also more complicated than for standard drugs, particularly if alternative routes such as oral administration are considered. And then there is cost, which is still relatively high compared to small molecules or more established biologics.

Specificity is also an issue. These molecules do not always stay where we want them to, and they can interact with unintended targets, leading to off-target or even undesired on-target effects [380]. On top of that, tumor cells are not static. They can adapt—for instance, by altering miRNA response elements or expressing mRNA isoforms with shorter 3′UTRs—which reduces the impact of miRNA-mediated regulation [381,382,383].

Still, things are moving forward. Improvements in oligonucleotide chemistry and delivery systems, such as lipid nanoparticles or targeted conjugates, are starting to make these approaches more feasible in a clinical context.

What is less clear is how well they will perform outside controlled settings. We still need more data from clinical studies on miRNA mimics, antagomiRs, and similar strategies to understand where they can really make a difference. At the same time, ncRNAs are likely to become increasingly useful as biomarkers, especially when combined into signatures that can help with patient stratification or prediction of treatment response.

To really move these approaches forward, we need a better understanding of how miRNAs, lncRNAs, and their targets interact in specific contexts. It is possible that integrated ncRNA profiling will eventually enter routine clinical practice, but we are not quite there yet.

Overall, there are still several open questions. At the same time, it is becoming clear that ncRNAs are changing how we look at cancer biology, and their potential relevance for both diagnosis and therapy is hard to dismiss.

## 9. Conclusions

Taken together, the evidence discussed here points to a relevant role of miRNA–lncRNA cross-regulation in gene expression control in cancer, even if its exact contribution is not always easy to pin down. These interactions rarely act as isolated events; more often, they are part of broader regulatory networks that connect post-transcriptional mechanisms with transcriptional and epigenetic layers.

The ceRNA hypothesis helped to frame this complexity at an early stage [6], but it has become clear that it does not capture the whole picture. Other mechanisms—such as RNA decay, changes in miRNA maturation, and chromatin-associated regulation—also seem to play a role, and likely do so in a context-dependent way.

From a functional standpoint, these networks intersect with several core aspects of cancer biology. They are linked to pathways like PI3K/AKT signaling, hypoxia–HIF responses, and epigenetic regulators such as EZH2, and through these connections they can influence proliferation, adaptation to stress, and metastatic behavior. That said, their effects are not consistent across contexts and appear to depend on factors such as transcript abundance, cell type, and microenvironmental conditions.

This context dependency makes these systems more difficult to study, but it also points to potential opportunities. While it complicates the identification of broadly applicable mechanisms, it may allow a more refined stratification of patients. In this regard, the relative stability of ncRNAs in biological fluids has supported their investigation as minimally invasive biomarkers. In particular, combined miRNA–lncRNA signatures may improve diagnostic and prognostic performance, although further validation is still required.

From a therapeutic standpoint, targeting ncRNA networks is an attractive strategy, since modulation of a single miRNA or lncRNA could potentially affect multiple downstream pathways. However, several challenges remain, including delivery efficiency, off-target effects, and immune-related toxicity [20]. In addition, the redundancy and combinatorial nature of RNA networks make it difficult to identify the most relevant therapeutic targets.

Future work will likely benefit from integration of multi-omics approaches with emerging technologies such as single-cell and spatial transcriptomics, which may help to better resolve these interactions within their native context.

Overall, miRNA–lncRNA cross-regulation adds another layer of complexity to cancer biology. Although several aspects remain to be clarified, a deeper understanding of these networks could contribute to improved current diagnostic strategies and to the development of RNA-based therapeutic approaches.

## Figures and Tables

**Table 1:** miRNA–lncRNA cross-regulatory axes across tumor types.

Cancer Type	lncRNA–miRNA Axis	Interaction	Biological Impact and Phenotype	Validated Regulatory Axis (lncRNA–miRNA–mRNA)	Experimental Validation	Ref.
Breast Cancer	HOTAIR–miR-129-5p	Sponge (ceRNA)	Increased Cell Proliferation	HOTAIR → miR-129-5p → FZD7 axis	in vitro	[121]
Triple-Negative Breast (TNBC)	MALAT1–miR-1	Sponge (ceRNA)	Cell-cycle progression and EMT-associated metastasis.	MALAT1 → miR-1 → EMT/cell-cycle regulatory genes	in vitro	[119]
Breast Cancer	UCA1–miR-18a	Sponge (ceRNA)	Apoptosis resistance and survival.	UCA1 → miR-18a → HIF1α axis	in vitro	[122]
TNBC	RMST–miR-4295	Sponge (ceRNA)	Autophagy and Ca^2+^ signaling under stress.	RMST → miR-4295 → ITPR1 (Ca^2+^ signaling)/mTOR axis	in vitro	[123]
Non-small-cell lung cancer (NSCLC)	MALAT1–miR-145-5p	Sponge (ceRNA)	Increased invasiveness and metastatic potential	MALAT1 → miR-145-5p → NEDD9 axis	in vitro	[124]
Lung	miR-217–MALAT1	Decay (Ago2)	EMT inhibition; lncRNA silencing	miR-217 → MALAT1 → EZH2-associated oncogenic axis	in vitro	[125]
NSCLC	DLX6-AS1–miR-144	Sponge (ceRNA)	Increased proliferation and invasiveness	DLX6-AS1 → miR-144 → PRR11 axis	in vitro + in vivo	[126]
Colorectal (CRC)	UCA1–miR-204-5p	Sponge (ceRNA)	Increased proliferation, migration, invasion and tumor growth	UCA1 → miR-204-5p → CREB1/BCL2/RAB22A axis	in vitro + in vivo	[127]
CRC	H19–miR-138/200a	Sponge (ceRNA)	Promotes epithelial–mesenchymal transition	H19 → miR-138/miR-200a → ZEB1/ZEB2 axis	in vitro + in vivo	[65]
CRC	MEG3–miR-141	ceRNA-like modulation	Increased oxaliplatin sensitivity.	MEG3 → miR-141 → PDCD4 axis	in vitro	[128]
Prostate (PCa)	UCA1–miR-184	Sponge (ceRNA)	Increased viability and reduced apoptosis	UCA1 → miR-184 → Bcl-2 axis	in vitro	[129]
PCa	PlncRNA-1–miR-34c/miR-297	ceRNA-like modulation	Enhanced cell survival and tumor growth	PlncRNA-1 → miR-34c/miR-297 → AR axis	in vitro	[130]
Hepatocellular carcinoma HCC	ATB–miR-200s	Sponge (ceRNA)	Enhanced EMT activation and tumor–stroma interaction.	ATB → miR-200 → ZEB1/ZEB2 axis (EMT); IL-11/STAT3 signaling (tumor–stroma crosstalk)	in vitro + in vivo	[131]
HCC	SNHG3–miR-214-3p	Sponge (ceRNA)	Promotes immune escape through regulation of PD-1 expression	SNHG3 → miR-214-3p → ASF1B → PD-1 axis	in vitro	[132]
HCC	miR-423-5p–MALT1	Decay (Ago2)	lncRNA down-modulation; reduced tumor progression and metabolic activity	miR-423-5p → MALAT1	in vitro	[11]
Head and Neck Squamous Cell Carcinoma (HNSCC)	LINC00668–miR-297	Sponge (ceRNA)	Promotes angiogenesis and tumor growth.	LINC00668 → miR-297 → VEGFA axis	in vitro	[133]
HNSCC	H19–miR-675-5p	Precursor	Stroma rewiring: CAF-mediated glycolysis.	H19 → miR-675-5p → PFKFB3 axis	in vitro + in vivo	[134]
Acute myeloid leukemia (AML)	NEAT1–miR-23a-3p	Sponge (ceRNA)	Increased cell proliferation and reduced apoptosis	NEAT1 → miR-23a-3p → SMC1A axis	in vitro	[135]

This table summarizes experimentally validated lncRNA–miRNA interactions reported across different tumor types and associated with key cancer processes, including proliferation, apoptosis resistance, epithelial–mesenchymal transition (EMT), metastasis, angiogenesis, immune evasion, metabolic adaptation, and therapy response. The main mode of interaction, the resulting biological effects, and the principal signaling pathways linked to each regulatory axis are indicated.

**Table 2:** Clinical landscape of ncRNA therapeutics and related RNA platforms in oncology.

Program/Agent	Modality/Delivery	Target and Class	Status (Trial ID)	Clinical Significance	Ref.
**MRX34**	miRNA mimic (dsRNA) in Liposomal NP (IV)	miR-34a (ncRNA/miRNA)	Phase I; Discontinued (NCT0182997)	First-in-human proof-of-concept for miRNA replacement; highlighted critical immune-mediated toxicity barriers.	[296,297]
**TargomiRs**	miRNA mimic in EGFR-targeted EDVs (IV)	miR-16 family (ncRNA/miRNA)	Phase I; Completed (NCT0236919)	Validated targeted delivery via bacterial minicells; demonstrated improved safety profile vs. non-targeted liposomes.	[302,303]
**Cobomarsen (MRG-106)**	LNA anti-miR (ASO); Intralesional/IV	miR-155 (oncomiR inhibition)	Phase II; Halted (NCT0258055)	Most advanced example of oncomiR antagonism in T-cell lymphomas; showed clinical activity before strategic termination.	[292]
**MTL-CEBPA**	saRNA in Lipid Nanoparticles (IV)	CEBPA upregulation (saRNA/RNAa)	Phase I/II; Ongoing (NCT02716012; NCT04710641)	Demonstrates the feasibility of transcriptional activation (RNAa) to reprogram the tumor myeloid microenvironment.	[304]
**BC-819**	Plasmid DNA (H19-driven DT-A); Intravesical	H19 promoter (lncRNA regulatory locus)	Phase IIb; Completed (NCT0039380)	Exploits lncRNA-specific expression as a “genetic switch” to drive targeted toxin-based tumor ablation.	[305,306]

**Table 3:** Comparative overview of miRNA-lncRNA interactions databases.

Database	Data Type	Prediction Strategy	Experimental Support	Key Strengths	Limitations	References
DIANA-LncBase	Predicted + Validated	miRanda, TargetScan features, CLIP-Seq integration	Yes	High-confidence scoring, frequent updates	Human-focused, limited non-model species	[341]
starBase	CLIP-Seq derived	AGO-binding site inference, CLIP-Seq integration	Yes	Low false-positive rate, ceRNA network support	Dependent on CLIP-Seq availability	[15]
miRcode	Predicted	Sequence complementarity	No	Genome-wide coverage, simple screening	No experimental validation	[94]
NPInter	Predicted + Validated	Literature curation, multi-method integration	Yes	Broad interaction types	Less specialized for miRNA-lncRNA	[342]
RNAInter	Predicted + Validated	Integrated computational pipelines	Yes	Comprehensive interactome coverage	Heterogeneous confidence metrics	[343]

## Data Availability

No new data were created or analyzed in this study. Data sharing is not applicable to this article.

## Abbreviations

AKT	Protein Kinase B
AML	acute myeloid leukemia
AR	androgen receptor
ASO	antisense oligonucleotide
ATG7	autophagy-related gene 7
BCL-family	B-cell lymphoma family
CAF	cancer-associated fibroblast
ccRCC	clear cell renal cell carcinoma
CDK	cyclin-dependent kinase
ceRNA	competing endogenous RNA
CML	chronic myeloid leukemia
CRC	colorectal cancer
CTCL	cutaneous T-cell lymphoma
CXCR4	C-X-C chemokine receptor type 4
DT-A	diphtheria toxin A
EDV	EnGeneIC Dream Vector
EGFR	epidermal growth factor receptor
EMT	epithelial–mesenchymal transition
EV	extracellular vesicle
E2F	E2 factor (transcription factor)
EZH2	enhancer of zeste homolog 2
FOSL2	FOS-like antigen 2
HCC	hepatocellular carcinoma
HDAC	histone deacetylase
HIF-1α	hypoxia-inducible factor 1-alpha
HNSCC	head and neck squamous cell carcinoma
IV	intravenous
ITPR1	inositol 1,4,5-trisphosphate receptor type 1
KDM5B	lysine demethylase 5B
LNA	locked nucleic acid
MAPK	mitogen-activated protein kinase
MCL1	myeloid cell leukemia 1
MMP16	matrix metalloproteinase 16
mTOR	mammalian target of rapamycin
ncRNA	non-coding RNA
NSCLC	non-small-cell lung cancer
PI3K	phosphoinositide 3-kinase
PULB	poly(U)-binding protein
RB	retinoblastoma protein
RISC	RNA-induced silencing complex
RNAa/saRNA	RNA activation/small activating RNA
SAE	serious adverse event
TFs	transcription factors
TNBC	triple-negative breast cancer
T-UCRs	transcribed ultraconserved regions
VEGF-A	vascular endothelial growth factor A
ZEB1/2	zinc finger E-box binding homeobox 1/2
